# Dietary Practices, Body Composition, and Sports Performance of Athletes

**DOI:** 10.3390/nu17193102

**Published:** 2025-09-30

**Authors:** Lewis A. Gough

**Affiliations:** Human Performance and Health Laboratory, Birmingham City University, Birmingham B15 3TN, UK; lewis.gough@bcu.ac.uk

Dietary practices can have a multitude of impacts on body composition, sporting performance, training adaptations, and health. If it is optimal, the aforementioned factors change favourably, and this has a large positive impact on the chances of achieving peak performance [1]. Whilst this is generally accepted, the impact of dietary intake on sport performance is poorly understood, and research consistently suggests that athletes engage in sub-optimal dietary practices [2]. Collectively, this editorial, *Dietary Practices, Body Composition and Sports Performance of Athletes*, provides a platform for researchers to disseminate novel data that help shape strategies to improve dietary practices with athletes. This brief editorial summarises some of these novel findings and provides a blueprint for future research.

Beginning with Martín-Rodríguez’s work (contribution 5) and their review of dietary practices, body composition, and sports performance, the authors suggest that nutrition strategies should be tailored on an individual level to best optimise sports performance and long-term health. The key target of the practitioner should be energy availability, as this is the key driver for performance, adaptation, and recovery, in agreement with previous research [3,4]. Research in specific sports such as rugby (contribution 8) (contribution 10) and football/soccer (contribution 6) also offer novel insight to energy availability. In a review, Roberts’s work (contribution 8) provides a useful framework for practitioners to help with achieving energy availability, sufficient macronutrient intake, and diet quality. This useful framework blends the academic literature and practical strategies to help practitioners in the field, and builds upon other research reporting dietary intake in rugby union players [5,6]. In an original research article, Soubani’s work (contribution 10) reports that young male rugby players meet the required protein and fat intakes during a micro-cycle of pre-season training. Their carbohydrate intake, however, was at the lower level of international recommendations. Some players were also below the recommended amount of iron, calcium, and vitamin D intake thresholds. This builds upon recent work that highlighted that more data is needed from youth rugby union players [7]. Similar to the recommendation of Martín-Rodríguez’s work (contribution 5), an individualised approach was suggested to ensure players can meet these nutritional targets. Finally, an important study in elite women footballers (contribution 6) also recommended targeted player education in the context of achieving energy availability and carbohydrate recommendations. Six players (34%) presented with low energy availability during a 10-day training camp. This important data from an elite population that builds upon other work in sub-elite and recreational female footballers [8]. Collectively, these studies report similar findings and recommendations, in that individualised strategies are important to ensure appropriate energy intake is achieved.

One question that is always proposed in the assessment of dietary practices is “how do we assess/screen for dietary intake and know it is valid?”—both Saenz’s work (contribution 9) and Buckley’ work (contribution 2) propose two new screening tools to help answer this common question in dancers and general athletes, respectively. Saenz’s work (contribution 9) is novel in that a new assessment tool specific to dance was used within a group of collegiate dance athletes (*n* = 33). In agreement with smaller studies in this area, they were alarmed at the low energy intake within this cohort. This leaves a gap for future work to address in terms of how this could be improved. The authors pose some suggestions including increasing food availability and time for food ingestion, increasing time for activity that increases muscle mass, dance-specific nutrition education, and increased body positivity language. This builds upon a recent study calling for a need for more intensive screening and support for dancing and nutrition [9]. In a development and validation study, (contribution 2) devised a new Athletic Disordered Eating (ADE) screening tool for current and former athletes. This 17-item screening tool focused on four sub-scales (food control, bingeing, body control, and body discontent) and reported an Internal Consistent Reliability of 0.91 and Intraclass Correlation Coefficient of 0.97. This tool can therefore be used widely across various sports and levels of competition. Notably, this screening tool is the first to be validated with former athletes, who are known to report disordered eating once retired [10,11]. It is important to note that a further study has supported the validity of this scale, by distributing the scale to an independent sample of *n* = 237 male and female elite athletes and reporting acceptable internal consistency, convergent and discriminant validity, and criterion-related validity [12].

In reference to supplement usage, Newbury’s work (contribution 7) investigated the supplement use in a UK high-performance swimming club across various age groups (ages 11–≥16 years of age). The authors report that younger athletes tend to decide on supplement choice due to parental guidance, which then transferred to the qualified sports nutritionist in the older age groups. This suggests that with younger age athletes parent/caregiver education is key to ensure the appropriate supplements are selected. This work builds upon the earlier findings of Mettler’s work [13] highlighting the low level of information young Swiss swimmers were accessing to inform their dietary supplement practices. In another study investigating supplement use, Christensen’s work (contribution 3) reports that the key influences on supplement choice were coaches and sponsorship agreements within a group of *n* = 25 adult elite ice hockey players. This may be concerning given the level of education provided to coaches on supplements and that sponsorship deals do not guarantee supplement quality. Interestingly, the ice hockey players also placed very little importance on the anti-doping education they were required to complete by their professional club. Some of these themes are consistent with a recent systematic review across multiple sports, suggesting that anti-doping represents a complex systemic problem where the support personnel, the coach, and the coach–athlete relationship represent key influences on the athletes’ decisions to dope [14]. This work highlights an area of future research into the effectiveness of anti-doping programmes on supplement use. Based on this evidence, the information athletes seek to inform supplement ingestion decisions is varied, and more work is needed to ensure that appropriately qualified individuals (i.e., nutritionists and dieticians) are consulted more often.

In endurance sports, two longitudinal studies in cycling (contribution 4) and cross-country skiing and biathletes Stenqvist’s work (contribution 11) investigated relative energy deficiency in sport (REDs) and bone health. In a one-year/season observation in professional endurance cyclists (*n* = 18), bone mineral density (BMD) reduced significantly in the legs, trunk, ribs, and pelvis. Generally, some athletes entered the season with borderline bone health, which has potential elevated risks for osteopenia or osteoporosis. In addition, this may increase the risk of fractures from cycling crashes, or exacerbated damage for any given crash versus those with better bone health [15]. The underlying mechanisms are suggested to be the lack of impact-loading, low energy availability (LEA), and calcium losses via sweat. This provides some nutritional considerations for future research that needs to investigate the interplay of loading activity/exercise and energy availability. In a longer study of three years, albeit in lesser trained Norwegian high-school cross-country skiers and biathletes (*n* = 13), similar themes were apparent with 38% reporting low lumbar BMD (Z-score ≤ −1). Many of the athletes had poor bone health at baseline, and most either lost or did not achieve expected pubertal bone mineral accrual. These findings suggest that, in high-school athletes (mean age: 16), the need for early screening and detection of impaired bone health is needed. Again, this highlights that more research is required on interventions to improve bone health in athletes, given the important role this has on body composition.

Finally, a novel review provided the nutritional considerations for elite golf (contribution 1). Given the unique nature of golf being classified as a predominantly skill-based sport, there are nutritional considerations to support cognitive performance, body composition, energy requirements, protecting immunity when travelling, and supplements. With distances of up to 20 km being covered and the time spent on the course ranging between approximately 4 and 8 h each day, the review suggested that energy intake is key to meet the high walking demands, as well as ensuring hydration is maintained throughout the round. There is also a possibility for certain nutritional ergogenic aids such as caffeine, since lower doses in the range of 3–5 mg·kg^−1^ body mass have been reported improve accuracy and drive distance. Moreover, given the potential of worldwide travel for elite golfers, maintaining usual food intake and food hygiene is vital to avoid illness. There may also be a role in maintaining vitamin D concentrations to support the immune system. This work has been used within a recent scoping review by O’Donnell and colleagues (2024) wherein similar inferences have been made, in addition to a call for more research specifically in golf [16].

In conclusion, this research topic has resulted in a significant contribution to knowledge that provides a blueprint for further research to improve our understanding of dietary practices and the associated impacts on body composition and sports performance in athletes.
